# Group B Streptococcus (GBS) Carriage in Pregnant Women: Possible Emergence of Rare Serotypes and Antibiotic Resistance in Neonatal Disease

**DOI:** 10.3390/microorganisms13071496

**Published:** 2025-06-26

**Authors:** Roberta Creti, Monica Imperi, Giovanni Gherardi, Giovanna Alfarone, Ilaria Marani, Caterina Vocale, Alberto Berardi, Serena Truocchio, Francesca Miselli

**Affiliations:** 1 Antibiotic Resistance and Special Pathogens Unit, Department of Infectious Diseases, Istituto Superiore di Sanità, 00161 Rome, Italy; monica.imperi@iss.it (M.I.); giovanni.gherardi@iss.it (G.G.); giovanna.alfarone@iss.it (G.A.); ilaria.marani@iss.it (I.M.); 2 Microbiology Unit, IRCCS Azienda Ospedaliero-Universitaria di Bologna, 40138 Bologna, Italy; caterina.vocale@aosp.bo.it; 3 Neonatal Intensive Care Unit, Department of Medical and Surgical Sciences of Mothers, Children and Adults, University of Modena and Reggio Emilia, 41125 Modena, Italy; alberto.berardi@unimore.it (A.B.); serena.truocchio@unimore.it (S.T.); misellifrancesca0@gmail.com (F.M.)

**Keywords:** *Streptococcus agalactiae*, group B streptococci, carriage, neonatal disease, serotype, antibiotic resistance

## Abstract

Maternal vaginal and rectal colonization by *Streptococcus agalactiae* (group B streptococcus, GBS) is the main risk factor for the development of newborn early-onset GBS disease (GBS-EOD). Much effort is in place for its prevention, including the development of vaccines. Currently, both a hexavalent glycoconjugate GBS vaccine against the most prevalent serotypes and a protein subunit vaccine have completed phase two clinical trials. GBS surveillance in both maternal carriage and neonatal disease is therefore important in establishing the coverage of the potential vaccines and in setting up the basis for pre- and post-marketing surveillance. A single-site study was conducted in the years 2020–2021 on the characteristics of 325 GBS strains (serotype distribution; identification of the alpha-like protein family member; and resistance to macrolides, tetracycline, and high-level gentamicin) isolated from the vaginal/rectal site in women in late pregnancy as well as in seven cases of GBS-EOD and one case of GBS-related stillbirth occurring in the same location and time period. The study indicated that the coverage of the developing vaccines was excellent (97.2% for the hexavalent glycoconjugate vaccine and 98.7% for the alpha-like protein subunit vaccine). However, the detection of the serotypes VI, VII, and IX—not covered by current vaccine formulations—accounting for 3.0% of isolates, as well as of negative alpha-like GBS strains from maternal carriage (1.2%), should be closely monitored over time. The high rates of GBS resistance to erythromycin (33.5%) and to clindamycin (29.5% in maternal carriage and 57.1% in GBS-EOD) was mostly due to the ever-increasing spread of the multidrug-resistant ST-17 subclone of serotype III. This finding, along with the newly emerging high-level gentamicin resistance in carriers (4.0%), mainly in serotype IV strains, poses a threat for the continued effectiveness of antibiotic therapy in invasive disease.

## 1. Introduction

*Streptococcus agalactiae* (group B streptococcus, GBS) is a β-hemolytic encapsulated Gram-positive bacterium that colonizes the human genitourinary and gastrointestinal tracts [1]. More than fifty years ago, GBS emerged as the leading infectious bacterial cause of invasive infections in newborns and infants. At present, GBS still remains the most frequent cause of meningitis and a prominent pathogen in sepsis in the first three months of life worldwide [2,3,4]. Conventionally, two forms of the disease are recognized: one with an early onset (EOD) within the first week of life and one with a late onset (LOD) within the first three months of life [5].

Ten immunologically distinct serotypes of GBS have been defined based on surface polysaccharides (Ia, Ib, and II to IX). A systematic review and meta-analysis including about 6500 strains from neonatal GBS disease isolated worldwide between the years 2000 and 2017 indicated that five serotypes, namely Ia, Ib, II, III, and V, accounted for more than 97% of all cases [6]. Serotype III strains are clinically the most important: they account for approximately 25% of colonising strains and 60% of strains causing invasive disease in neonates and infants, although geographical variation exists [7].

Alpha-like proteins (Alp) is a family of surface proteins constituted by different members (Alpha-C, Rib, Alp1, Alp2/3, and Alp4) made of an internal region of identical repeated units whose number variation modulates their role as important virulence factors mediating the adhesion and invasion of GBS [8].

GBS vaginal colonization is the main risk factor for the development of EOD by vertical transmission at delivery and can be prevented by the administration of an intrapartum antibiotic prophylaxis (IAP) [9,10,11,12]. The vertical transmission is confirmed by the similar serotype distribution of GBS strains isolated from colonized pregnant women and those causing disease in newborns [13,14].

To this aim, it is important to investigate the characteristics of GBS strains from maternal colonization to promptly detect emergent serotypes and antibiotic resistance traits that could affect the newborn disease. This is particularly important considering that GBS vaccine formulations are in development and a pre-market surveillance is important for assessing the coverage and cost–benefit upon their implementation [15,16,17,18,19,20]. Currently, two formulations are in clinical trials: a hexavalent glyconjugate vaccine containing GBS serotypes Ia, Ib, II, III, IV, and V, and a protein subunit vaccine composed of a fusion protein made by the N-terminal portion of the alpha-like surface protein family (Alpha-C, Rib, and Alp1-3) [20,21]. Similarly, a post-market surveillance can monitor any changes in the expected efficacy of the vaccination. Moreover, the analysis in maternal colonization can anticipate the emergence of escape-vaccine GBS strains and antibiotic resistance that may eventually manifest in newborn disease.

Here we present a single site-based study on the serotype and antibiotic susceptibility profile of GBS strains from carrier pregnant women collected during the years 2020–2021. GBS-EOD cases that occurred in the same time period were also considered for microbiological characterization as sentinels of the speed of transmission and diffusion of emergent serotypes and antibiotic resistance traits.

## 2. Materials and Methods

### 2.1. Women Enrollment and Bacterial Collection

Vaginal–rectal swabs were collected from women in late pregnancy (36–37 weeks of gestation) by an appointed gynecologist at the Department of Medical and Surgical Sciences for Mother, Child and Adult and sent to the Clinical Microbiology Unit of University Hospital of Modena. The samples were processed according to the CDC recommendations for GBS detection and identification [22]. The time period of collection was from 15 July 2020 to 2 November 2021.

Demographic and clinical characteristics were collected according to a standardized form. The women were prospectively followed until delivery.

The GBS isolates from colonized women as well as from neonatal GBS disease (occurring in the same time period) were sent to the National Reference Centre for Streptococci at the National Public Health Institute (ISS-NRL). Species confirmation was done by the determination of group B Lancefield surface antigen using the streptococcal grouping kit (Oxoid). Serotyping was based on the latex agglutination test using the ImmuLexTM StrepB-Kit (SSI Diagnostica, Hillerød, Denmark). Molecular typing of capsular types Ia–IX was performed using a multiplex polymerase chain reaction (PCR) assay, both in the case of phenotypically not-typeable strains (those with discrepant serotype results when tested in duplicate) and for confirming the results of the agglutination test, with a 100% agreement [13,14]. The identification of the Alpha-like (Alp) surface proteins family was performed using a multiplex PCR [13,14]. The antimicrobial resistance profiles to erythromycin, clindamycin, and tetracycline were performed as already described [13,14]. High-level gentamycin resistance (HLGR) was assessed both phenotypically by disk-diffusion (MIC > 1024 ng/µL) and genotypically for the presence of an intact *aac(6’)-Ie-aph(2’’)*-Ia gene [23].

Serotype III strains were assessed for the presence of the *hvg*A gene for the identification of the hypervirulent ST-17 lineage [24]. Pilus island gene content was performed by a PCR assay that identified the presence of pilus island (PI)-1, PI-2a, and PI-2b [25].

### 2.2. Ethical Approval

The enrolment of pregnant women was part of the Work Package 3 task of the PREPARE Project [26]. The study received approval from the Modena University Hospital Ethics Committee (prot N° 0011051/20 on 20 April 2020). All enrolled women and parents of infected neonates signed an informed consent.

## 3. Results

### 3.1. Socio–Demographic and Clinical Characteristics of the Studied Group

The majority of GBS-colonized women (63.7%) delivered at an age between 30 and 39 years and were of Caucasian origin (70.1%) (Table 1). All but one had a singleton pregnancy and 39.4% were about to give birth to their first baby.

Vaginal delivery was the most common (81.2%). In addition to GBS colonization, obstetric risk factors at birth were present in 52 deliveries (16%), the vast majority of which (48 cases, 92.3%) exhibited only one risk factor. In particular, risk factors included preterm labor (3 women), preterm labor and prolonged rupture of membranes (2 women), fever (8 women), fever and prolonged rupture of membranes (2 women), prolonged rupture of membranes (33 women), and a previous baby with GBS infection (4 women) (Table 2).

281 women (86.5%) received IAP, although all were eligible to receive it because of their GBS colonization status. However, 36 women out of 41 who did not receive IAP underwent cesarean section; no information was available on the integrity of membranes (intact amniotic membranes do not require IAP administration).

The duration of IAP was reported in 275 cases and it was adequate (i.e., initiated 4 h or more before birth) in 79 cases (28.7%).

Ampicillin was the antibiotic of first choice in IAP in 93.6% of cases. In the remaining cases, clindamycin was offered.

No newborn developed an invasive GBS-EOD disease.

### 3.2. Serotype Distribution

**Figure 1:**
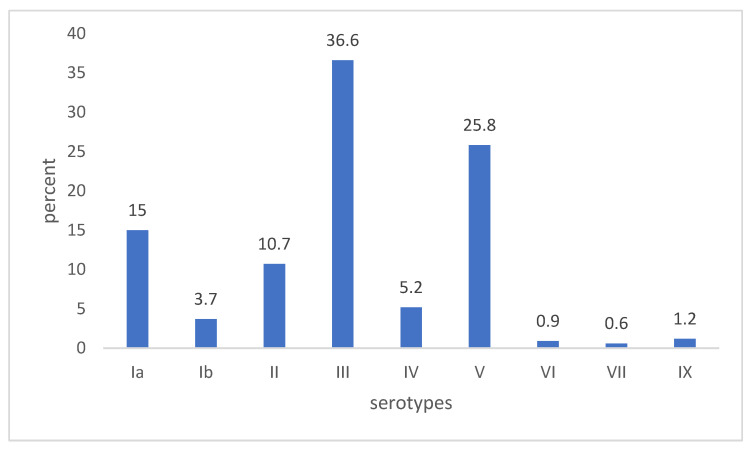
Distribution and frequency of serotypes identified in 325 GBS strains from pregnant women.

The prevalent GBS serotypes among pregnant women were serotype III (36.6%), serotype V (25.8%), and serotype Ia (15.0%) (Figure 1). Serotype IV represented 5.2% of strains. Serotypes VI, VII, and IX, not present in the glycoconjugate vaccine formulation under development, accounted for 3.0% of isolates (9 out of 325).

### 3.3. Distribution of the Alpha-like Protein Family Members

All but four GBS isolates possessed an Alp member. Of the GBS strains, only serotype V did not possess any Alp member. The most prevalent Alp member was Rib (34.7%), followed by Alp1 (22.4%), alpha-C (22.8%), and Alp2/3 (19.7%). An association between the Alp member and serotype was observed: the Rib protein with serotype III, the Alpha-C member with serotype Ib and serotype IV, and the Alp1 member with serotype Ia (Table 3).

### 3.4. Antibiotic Susceptibility

Constitutive resistance to both erythromycin and clindamycin was displayed by 73 maternal strains (22.4%) that possessed the *erm*B genetic determinant, followed by the inducible clindamycin resistance (21 strains, 6.4%) determined by the *erm*A gene and by the M phenotype (erythromycin resistance and clindamycin susceptibility; 15 strains, 4.6%) encoded by the *mef*A gene (Table 4). Two serotype III strains displayed the unusual CRES phenotype (resistance to clindamycin and susceptibility to erythromycin). Overall resistance to erythromycin was then 33.5% (109 out of 325 isolates), while resistance to clindamycin was 29.5% (96 out of 325 isolates). The relationship between serotypes and antibiotic resistance in GBS carrier strains is reported in Table 4. Almost half of serotype V isolates; about one-third of serotypes Ia, Ib, II, and III; and a quarter of serotype IV displayed resistance to macrolides. Constitutive resistance was distributed among all resistant serotypes; on the contrary, inducible clindamycin resistance was restricted to serotypes Ia, II, III, and V and the M phenotype to serotypes Ia, Ib, and V. Most erythromycin-resistant serotype III strains (28/34, 82.3%) belonged to the hypervirulent ST17 lineage by possessing the gene encoding the invasin HvgA. Given the presence of both the *erm*B and *tet*O genes along with the loss of pilus island 1 in 71.4% (20/28) of these ST-17-resistant strains, we can confidently attribute them to the emerging multidrug resistant (MDR) ST-17 sublineage, worldwide diffused and ever-increasing in our country since the year 2015 [14,27,28].

Tetracycline resistance was expressed by 89.2% of maternal strains (290/325).

It is noteworthy that high-level gentamicin resistance (HLGR) was increasingly detected in the maternal GBS collection, reaching up to 4.0% (13 out of 325 strains). HLGR was mainly due to an emergent serotype IV clonal lineage [29] and to few strains of serotypes II and V (Table 4).

Notably, in addition to the MDR ST-17 sublineage, two isolates of serotype V and one of serotype II were also multidrug-resistant (at least three antibiotic classes, MDR), as they showed resistance to all the antimicrobials tested (macrolides/lincosamides, tetracycline, and gentamicin).

### 3.5. GBS Newborn Cases

Seven EOD cases and one GBS stillbirth occurred during approximately the same time period, from mid-year 2020 to spring 2022. The affected babies were not born to women in the study group; however, the cases were typed as they were related to local circulation of GBS strains. As for the GBS maternal strains, serotype III was prevalent (five cases, all possessing the Rib protein). One EOD case was caused by serotype Ia (Alp1 protein) and one by serotype IV (Alpha-C protein). The GBS stillbirth was caused by serotype V (Alp2/3 protein).

Four GBS-EOD cases and the GBS stillbirth were caused by macrolide-resistant strains (three serotype III, one serotype Ia, and one serotype V). All expressed the constitutive resistance to both erythromycin and clindamycin determined by the presence of the *erm*B gene. The three neonatal serotype III strains belonged to the MDR ST-17 sublineage.

Tetracycline resistance was expressed by 85.7% of GBS-EOD strains (six out of seven).

No HLGR was detected among the GBS-EOD strains.

## 4. Discussion

The purpose of this study was to analyze the serotype and Alp protein of GBS strains from carriers to assess the potential coverage of the developing vaccines as well as to evaluate the possible emergence of particular serotypes and/or antibiotic resistance traits that could impact the management and treatment of GBS-EOD. Moreover, GBS carriers in late pregnancy were identified and prospectively followed until delivery to verify compliance with prevention policies.

Overall, 16% of GBS carriers under study presented with at least one obstetric risk factor for EOD in addition to GBS colonization at delivery. This proportion was in line with that (13.4%) reported in the study by the pan-European DEVANI (Design of a Vaccine Against Neonatal Infections) consortium conducted in the years 2008–2011 on 1083 pregnant GBS-colonized women [30], demonstrating that this aspect has not changed over time.

Adherence to IAP was high and, even though only about one-fourth of women received an adequate IAP (administered four or more hours before birth), no newborn developed an invasive GBS-EOD disease. This finding reinforces the need to revise the concept of adequate and inadequate IAP considering that levels above the minimal inhibitory concentration (MIC) for GBS in both neonatal bloodstream and the amniotic fluid are also obtained when IAP is administered for less than four hours [31].

The rate of GBS carriage in pregnant women was not included in the study objectives; therefore no comparisons with available national and/or site-based data could be made [32,33,34].

Serotype III was the most prevalent (36.6%), followed by serotypes V (25,8%), Ia (15%), and II (10.7%). This distribution was in line with previous national data [13,34] as well with those from the DEVANI study that identified serotype III (33.6%) as the predominant serotype, followed by serotype Ia (20.2%), serotype V (18.4%), and serotype II (13.9%) [30]. The predominance of serotype III has already been reported. A systematic review and meta-analysis that included serotyping data of nearly seventeen thousand GBS isolates from pregnant women in different parts of the world during the years 2005–2016 indicated that the serotypes Ia to V represented 98% of the isolates. Serotype III accounted for 25% of total isolates, although less frequently in some South American and Asian countries [4]. Similar findings were also reported by a global review covering the period 2001–2018 which indicated that serotype III was the most frequent (25%), along with Ia and V (both approximately 20%) in several regions of the world (Europe and northern America, eastern Asia, southern, eastern/central Africa, and Australia/New Zealand) [35].

The emergence of the rare serotype IV, representing 5.2% of the total GBS colonizing isolates in our study, has been a novel finding in our country but in line with what has been reported in Europe and northern America, while its diffusion in southern Africa is lower (about 3%) [36].

Non-vaccine serotypes VI, VII, and IX accounted for 3.0% of the total strains in our study, while serotypes VII and VIII were not identified. Serotypes VI to IX have been reported very rarely in Europe and North America. However, in Denmark, serotype IX was the most frequent (21%), followed by serotype III (19%), in a selected set of carriage GBS isolates from women in labor [36].

A high carriage rate of serotype IX among pregnant women has been also observed in non-European countries, such as Ghana [37] and Argentina [38]. In south-eastern Asia, eastern Asia, southern Asia, and western Africa, serotypes VI, VII, VIII, and IX have been reported more commonly [6,35,37,39,40]. Serotype VI was highly prevalent among asymptomatic pregnant women admitted for labor in 2017 in a single center in Israel (40.8%) [41]. High rates of serotype VI were also identified among pregnant women in Japan (9.5%) [42], Malaysia (22.3%) [43], and Egypt (12.2%) [44].

In general, the distribution of serotypes in pregnant women mirrors that of serotypes in GBS-EOD, confirming that bacterial vertical transmission during delivery is the main cause of newborn invasive disease. Indeed, a systematic review and meta-analysis that included about 6500 neonatal GBS strains isolated worldwide from EOD between the years 2000 and 2017 indicated that in maternal carriage, five serotypes, namely Ia, Ib, II, III, and V, accounted for more than 97% of all cases. Serotype III strains were clinically the most important and the most frequent in almost all continents [6]. Accordingly, the DEVANI study found that serotypes Ia, Ib, II, III, and V accounted for 93.9% of GBS-EOD strains [7].

In this study, the rates of resistance to erythromycin and clindamycin were 33.5% and 29.5%, respectively. These rates were higher than those reported in our previous investigations including GBS isolates from invasive diseases in neonates and in non-pregnant adults (28% and 26.8%, respectively, for erythromycin and 28.8% and 24.1% for clindamycin) over the period 2015–2019 [14,45].

The resistance to erythromycin of GBS strains under study was associated with serotype V (45.2%), followed by serotypes Ia (36.7%), Ib (33.3%), III (28.6%), II (28.6%), and IV (23.5%). The association between the resistance to erythromycin and serotype V has been already noted, independently from the source of isolation [46], while that with serotype III is emerging due to the spread of the multidrug resistant (MDR) ST17 subclone detected starting from the year 2015 in our country [14,27,28,46,47]. In this study, the MDR-ST17 subclone represented 6.1% of carrier GBS strains and, consequently, this clone is also assuming an increasingly predominant role in neonatal infections in our country [14].

The high resistance rates to erythromycin and clindamycin were slightly higher or similar to those observed in other European studies of GBS-colonized pregnant women collected from 2015 onwards, with erythromycin and clindamycin resistance rates ranging between 21 and 35% and 22 and 35%, respectively [36,48,49,50].

In Italy, a study from Eastern Sicily in the years 2015–2019 on GBS carriage showed resistance rates to erythromycin and clindamycin of 40% and 30%, respectively [34].

Penicillin or ampicillin are the antibiotics of choice for IAP in the prevention GBS-EOD infection [51]. Clindamycin may be the second-line antibiotic in penicillin-allergic women, due to its ability to cross the placental barrier and to reach higher concentrations in the cord blood [52]. Nevertheless, considering the alarming rate of clindamycin resistance in GBS, the UK National Institute for Health and Care Excellence (NICE) (www.nice.org.uk/guidance/ng195, accessed on 25 May 2025) has changed the recommendation to use clindamycin as an alternative for penicillin-allergic women in IAP. In contrast, in the United States, clindamycin is still the antibiotic of second choice in the case of women allergic to penicillin with a high risk of anaphylaxis. However, the American Congress of Obstetricians and Gynecologists (ACOG) guidelines report that the two prenatal assessments most commonly omitted are the determination of the nature of the penicillin allergy and the evaluation of susceptibility of a GBS isolate to clindamycin [12].

In the absence of Italian guidelines addressing the antibiotic of choice for IAP in women at high risk of anaphylaxis, our survey indicated that the use of clindamycin should be carefully considered if an antibiotic susceptibility test is not available, because in one case out of three, the GBS strain colonizing the parturient was clindamycin-resistant. Indeed, in our study, 20 out of 321 women received clindamycin as IAP and 5 of them were colonized by a clindamycin-resistant GBS strain. Luckily, the newborns did not developed GBS-EOD.

The resistance rate to tetracycline was 89.2%, in line with national and international data confirming the high diffusion of this resistance determinants among human GBS strains [14,45,53].

HLGR is an emergent antibiotic resistance in GBS whose clinical significance is still not clear. Gentamicin is not an antibiotic used in case of GBS infection but it is administered in combination with ampicillin as initial empiric therapy for neonatal sepsis and meningitis because of the enhanced bactericidal activity [54,55]. High-level aminoglycoside resistance abrogates the enhanced bactericidal activity and this can also constitute an advantage for GBS. We recently reported that the emergence of HLGR in GBS in our country is linked to the emergence of the serotype IV ST1010 (CC452) clonal lineage associated with the acquisition of a novel integrative and conjugative element containing the HLGR gene [29].

In our study, the most prevalent alpha-like surface protein was Rib (34.7%), followed by Alp1 (22.4%), alpha-C (22.8%), and Alp2/3 (19.7%). Our survey identified specific serotype/alpha-like protein associations, namely serotype III/Rib, serotype Ib/Alpha-C, serotype IV/Alpha-C, and serotype Ia/Alp1. These associations have been already widely reported, indicating that the alpha-like surface proteins are highly conserved within a given serotype [14,15].

The World Health Organization (WHO) has underlined the development of a GBS vaccine as a priority need [56]. The developing hexavalent glycoconjugate vaccine targeting serotypes Ia, Ib, II, III, IV, and V has the potential to prevent 95% of the colonizing isolates worldwide [35]. In this study, the six serotypes included in the hexavalent vaccine accounted for 97.2% of the total GBS maternal isolates. Similarly, the developing protein subunit vaccine would have a potential coverage of 98.7%.

This study had some limitations. First, it was a monocentric study and therefore did not represent the epidemiological characteristics of maternal GBS colonization at the national level, considering that the GBS serotype distribution and resistance rate may vary depending on the geographic area. Second, the study was not designed to systematically perform the vaginal/rectal culture at the antenatal screening, so colonization rates could not be inferred. Third, the possible co-carriage of mixed populations of GBS isolates was not investigated, although this is not uncommon [57,58,59]. Fourth, except for ST-17, genetic clonal diversity of GBS isolates was not characterized.

The hypothesis that the detection of particular microbiological characteristics in maternal GBS colonization may anticipate those in GBS-EOD strains was supported by the fact that, following the increase in serotype IV in carriers observed in this study, a case of GBS-EOD caused by a HLGR serotype IV was first reported to our national surveillance during the same period as this study, but not in the same area. In addition, two serotype IV GBS-EOD cases from the site of this study were subsequently reported to our national surveillance in the years 2023 and 2025. One of these was HLGR.

These findings reinforce the need for integrated surveillance of GBS strains from colonization and disease to ensure the optimal management and treatment of GBS-EOD.

## Figures and Tables

**Table 1:** Socio–demographic characteristics of the studied group.

**Age groups (years)**	
<20	0
20–29	30
30–39	207
>40	88
**Ethnicity**	
African	27
Asian	16
North-African	43
South American	11
Caucasian	228
Not reported	3
**Gravidity**	
Primigravida	128
Multigravida	194
Not reported	3
**Pregnancy type**	
Singleton pregnancy	324
Multiple pregnancy	1

**Table 2:** Clinical characteristics of GBS-colonized women.

**Mode of delivery**	
Vaginal	264
Elective cesarean section	59
Emergency cesarean section	None
Not reported	2
**Preterm labor (<37 weeks)**	
Yes	5
No	318
Not reported	2
**Maternal febrile syndrome**	
Yes	10
No	313
Not reported	2
**Rupture of amniotic membranes > 18 h**	
Yes	37
No	284
Not reported	4
**GBS bacteriuria**	
Yes	None
No	325
**Previous neonatal GBS infection**	
Yes	4
No	295
Not reported	26
**IAP administration**	
Yes	281
No	41
Not reported	3

**Table 3:** Relationship between GBS serotype and Alp member from pregnant women.

Serotype (N)	Alp Member (N, %)
Ia (49)	Alp1 (38, 77.5%); Alpha-C (4, 8.2%), Alp2/3 (4, 8.2%), Rib (3, 6.1%)
Ib (12)	Alpha-C (8, 66.7%), Alp2/3 (3, 25.0%), Alp1 (1, 8.3%)
II (35)	Rib (13, 37.1%), Alpha-C (12, 34.3%), Alp1 (6, 17.1%), Alp2/3 (4, 11.4%)
III (119)	Rib (95, 79.8%), Alp2/3 (21, 17.7%) Alpha-C (3, 2.5%)
IV (17)	Alpha-C (10, 58.8%), Alp1 (6, 35.3%), Alp2/3 (1, 5.9%)
V (84)	Alp2/3 (29, 34,5%), Alpha-C (27, 32.1%), Alp1 (22, 26.2%), Rib (2, 2,4%), negative (4, 4.8%)
VI (3)	Alpha-C (3)
VII (2)	Alp2/3 (2)
IX (4)	Alpha-C (4)

**Table 4:** Relationship between serotype and antibiotic resistance profile in GBS strains from pregnant women.

Serotype	Erythromycin Resistance	Macrolide Resistance Phenotype	Tetracycline Resistance	HLGR
Ia (49)	18 (36.7%)	CR (3); IR (2); M (13)	42 (85.7%)	0
Ib (12)	4 (33.3%)	CR (3); IR (0); M (1)	10 (83.3%)	0
II (35)	10 (28.6%)	CR (9); IR (1); M (0)	31 (88.6%)	1 (2.8%)
III (119) ^a^	34 (28.6%)	CR (27); IR (7); M (0); CRES (2)	112 (94.1%)	0
IV (17)	4 (23.5%)	CR (4); IR (0); M (0)	8 (47%)	9 (52.9%)
V (84)	38 (45.2%)	CR (26); IR (11); M (1)	82 (97.6%)	3 (3.6%)
VI (3)	1	CR (1); IR (0); M (0)	1	0
VII (2)	0	CR (0); IR (0); M (0)	2	0
IX (4)	0	CR (0); IR (0); M (0)	2	0
**Total**				
325	109 (33.5%)	CR (73); IR (21); M (15); CRES (2)	290 (89.2%)	13 (4%)

CR: constitutive resistance to both erythromycin and clindamycin; IR: resistance to erythromycin and inducible resistance to clindamycin; M: resistance to erythromycin and susceptibility to clindamycin; CRES: resistance to clindamycin and susceptibility to erythromycin; HLGR: high-level gentamicin resistance; ^a^ strains belonged to the MDR ST-17 sublineage.

## Data Availability

The original contributions presented in the study are included in the article. Further inquiries can be directed to the corresponding author.

## References

[1] Ling J., Hryckowian A.J. (2024). Re-framing the importance of Group B *Streptococcus* as a gut-resident pathobiont. Infect. Immun..

[2] Coggins S.A., Puopolo K.M. (2024). Neonatal Group B Streptococcus Disease. Pediatr. Rev..

[3] Seale A.C., Bianchi-Jassir F., Russell N.J., Kohli-Lynch M., Tann C.J., Hall J., Madrid L., Blencowe H., Cousens S., Baker C.J. (2017). Estimates of the burden of group B streptococcal disease worldwide for pregnant women, stillbirths, and children. Clin. Infect. Dis..

[4] Lawn J.E., Bianchi-Jassir F., Russell N.J., Kohli-Lynch M., Tann C.J., Hall J., Madrid L., Baker C.J., Bartlett L., Cutland C. (2017). Group B Streptococcal Disease Worldwide for Pregnant Women, Stillbirths, and Children: Why, What, and How to Undertake Estimates?. Clin. Infect. Dis..

[5] Baker C.J. (2013). The spectrum of perinatal group B streptococcal disease. Vaccine.

[6] Madrid L., Seale A.C., Kohli-Lynch M., Edmond K.M., Lawn J.E., Heath P.T., Madhi S.A., Baker C.J., Bartlett L., Cutland C. (2017). Infant Group B Streptococcal Disease Incidence and Serotypes Worldwide: Systematic Review and Meta-analyses. Clin. Infect. Dis..

[7] Lohrmann F., Hufnagel M., Kunze M., Afshar B., Creti R., Detcheva A., Kozakova J., Rodriguez-Granger J., Sørensen U.B.S., Margarit I. (2023). Neonatal invasive disease caused by *Streptococcus agalactiae* in Europe: The DEVANI multi-center study. Infection.

[8] Lachenauer C.S., Creti R., Michel J.L., Madoff L.C. (2000). Mosaicism in the alpha-like protein genes of group B streptococci. Proc. Natl. Acad. Sci. USA.

[9] Russell N.J., Seale A.C., O’Sullivan C., Le Doare K., Heath P.T., Lawn J.E., Bartlett L., Cutland C., Gravett M., Ip M. (2017). Risk of Early-Onset Neonatal Group B Streptococcal Disease with Maternal Colonization Worldwide: Systematic Review and Meta-analyses. Clin. Infect. Dis..

[10] Berardi A., Spada C., Creti R., Ambretti S., Chiarabini R., Barozzi A., Pagano R., Sarti M., Pedna M.F., Fornaciari S. (2020). Risk factors for group B streptococcus early-onset disease: An Italian, area-based, case-control study. J. Matern. Fetal Neonatal Med..

[11] Le Doare K., O’Driscoll M., Turner K., Seedat F., Russell N.J., Seale A.C., Heath P.T., Lawn J.E., Baker C.J., Bartlett L. (2017). GBS Intrapartum Antibiotic Investigator Group. Intrapartum Antibiotic Chemoprophylaxis Policies for the Prevention of Group B Streptococcal Disease Worldwide: Systematic Review. Clin. Infect. Dis..

[12] (2020). Prevention of Group B Streptococcal Early-Onset Disease in Newborns: ACOG Committee Opinion, Number 797. Obstet. Gynecol..

[13] Creti R., Imperi M., Berardi A., Pataracchia M., Recchia S., Alfarone G., Baldassarri L., Italian Neonatal GBS Infections Working Group (2017). Neonatal Group B Streptococcus Infections: Prevention Strategies, Clinical and Microbiologic Characteristics in 7 Years of Surveillance. Pediatr. Infect. Dis. J..

[14] Creti R., Imperi M., Berardi A., Lindh E., Alfarone G., Pataracchia M., Recchia S., The Italian Network on Neonatal And Infant Gbs Infections (2021). Invasive Group B Streptococcal Disease in Neonates and Infants, Italy, Years 2015–2019. Microorganisms.

[15] Trotter C.L., Alderson M., Dangor Z., Ip M., Le Doare K., Nakabembe E., Procter S.R., Sekikubo M., Lambach P. (2023). Vaccine value profile for Group B streptococcus. Vaccine.

[16] Kokori E., Olatunji G., Komolafe R., Ogieuhi I.J., Oyebiyi B., Ajayi I., Muogbo I., Ukoaka B., Samuel O., Aderinto N. (2024). Maternal GBS vaccination for preventing group B streptococcus disease in newborns: A mini review of current evidence. Int. J. Gynaecol. Obstet..

[17] Vekemans J., Crofts J., Baker C.J., Goldblatt D., Heath P.T., Madhi S.A., Le Doare K., Andrews N., Pollard A.J., Saha S.K. (2019). The role of immune correlates of protection on the pathway to licensure, policy decision and use of group B Streptococcus vaccines for maternal immunization: Considerations from World Health Organization consultations. Vaccine.

[18] Bjerkhaug A.U., Ramalingham S., Mboizi R., Le Doare K., Klingenberg C. (2024). The immunogenicity and safety of Group B Streptococcal maternal vaccines: A systematic review. Vaccine.

[19] Thorn N., Guy R.L., Karampatsas K., Powell M., Walker K.F., Plumb J., Khalil A., Greening V., Eccleston E., Trotter C. (2024). GBS vaccines in the UK: A round table discussion. F1000Res.

[20] Pena J.M.S., Lannes-Costa P.S., Nagao P.E. (2024). Vaccines for *Streptococcus agalactiae*: Current status and future perspectives. Front. Immunol..

[21] Le Doare K., Benassi V., Cavaleri M., Enwere G., Giersing B., Goldblatt D., Heath P., Hombach J., Isbrucker R., Karampatsas K. (2025). Clinical and regulatory development strategies for GBS vaccines intended for maternal immunisation in low- and middle-income countries. Vaccine.

[22] Filkins L., Hauser J.R., Robinson-Dunn B., Tibbetts R., Boyanton B.L., Revell P. (2020). American Society for Microbiology Provides 2020 Guidelines for Detection and Identification of Group B *Streptococcus*. J. Clin. Microbiol..

[23] Creti R., Imperi M., Berardi A., Angeletti S., Gherardi G. (2022). Laboratory breakpoints for assessing high level gentamicin resistance in *Streptococcus agalactiae*: It is the time for a consensus. Clin. Microbiol. Infect..

[24] Lamy M.C., Dramsi S., Billoët A., Réglier-Poupet H., Tazi A., Raymond J., Guérin F., Couvé E., Kunst F., Glaser P. (2006). Rapid detection of the “highly virulent” group B Streptococcus ST-17 clone. Microbes Infect..

[25] Springman A.C., Lacher D.W., Waymire E.A., Wengert S.L., Singh P., Zadoks R.N., Davies H.D., Manning S.D. (2014). Pilus distributionamong lineages of group b streptococcus: An evolutionary and clinical perspective. BMC Microbiol..

[26] Berardi A., Cassetti T., Creti R., Vocale C., Ambretti S., Sarti M., Facchinetti F., Cose S., Heath P., The Prepare Network (2020). The Italian arm of the PREPARE study: An international project to evaluate and license a maternal vaccine against group B streptococcus. Ital. J. Pediatr..

[27] Campisi E., Rosini R., Ji W., Guidotti S., Rojas-López M., Geng G., Deng Q., Zhong H., Wang W., Liu H. (2016). Genomic Analysis Reveals Multi-Drug Resistance Clusters in Group B Streptococcus CC17 Hypervirulent Isolates Causing Neonatal lnvasive Disease in Southern Mainland China. Front. Microbiol..

[28] Martins E.R., Pedroso-Roussado C., Melo-Cristino J., Ramirez M., Portuguese Group for the Study of Streptococcal Infections (2017). Streptococcus agalactiae Causing Neonatal Infections in Portugal (2005–2015): Diversification and Emergence of a CC17/PI-2b Multidrug Resistant Sublineage. Front. Microbiol..

[29] Creti R., Imperi M., Khan U.B., Berardi A., Recchia S., Alfarone G., Gherardi G. (2024). Emergence of High-Level Gentamicin Resistance in *Streptococcus agalactiae* Hypervirulent Serotype IV ST1010 (CC452) Strains by Acquisition of a Novel Integrative and Conjugative Element. Antibiotics.

[30] Lohrmann F., Efstratiou A., Sørensen U.B.S., Creti R., Decheva A., Křížová P., Kozáková J., Rodriguez-Granger J., De La Rosa Fraile M., Margarit I. (2025). Maternal *Streptococcus agalactiae* colonization in Europe: Data from the multi-center DEVANI study. Infection.

[31] Berardi A., Spada C., Vaccina E., Boncompagni A., Bedetti L., Lucaccioni L. (2020). Intrapartum beta-lactam antibiotics for preventing group B streptococcal early-onset disease: Can we abandon the concept of ‘inadequate’ intrapartum antibiotic prophylaxis?. Expert Rev. Anti Infect. Ther..

[32] Finale E., Spadea T., Mondo L., Arnulfo A., Capuano A., Ghiotti P., Barbaglia M., Guala A. (2022). *Streptococcus agalactiae* in pregnancy and the impact of recommendations on adherence to guidelines: An Italian area-based study. J. Matern. Fetal Neonatal Med..

[33] Serra G., Lo Scalzo L., Giordano M., Giuffrè M., Trupiano P., Venezia R., Corsello G. (2024). Group B streptococcus colonization in pregnancy and neonatal outcomes: A three-year monocentric retrospective study during and after the COVID-19 pandemic. Ital. J. Pediatr..

[34] Genovese C., D’Angeli F., Di Salvatore V., Tempera G., Nicolosi D. (2020). *Streptococcus agalactiae* in pregnant women: Serotype and antimicrobial susceptibility patterns over five years in Eastern Sicily (Italy). Eur. J. Clin. Microbiol. Infect. Dis..

[35] Bianchi-Jassir F., Paul P., To K.N., Carreras-Abad C., Seale A.C., Jauneikaite E., Madhi S.A., Russell N.J., Hall J., Madrid L. (2020). Systematic review of Group B Streptococcal capsular types, sequence types and surface proteins as potential vaccine candidates. Vaccine.

[36] Slotved H.C., Møller J.K., Khalil M.R., Nielsen S.Y. (2021). The serotype distribution of *Streptococcus agalactiae* (GBS) carriage isolates among pregnant women having risk factors for early-onset GBS disease: A comparative study with GBS causing invasive infections during the same period in Denmark. BMC Infect. Dis..

[37] Slotved H.C., Dayie N.T.K.D., Banini J.A.N., Frimodt-Møller N. (2017). Carriage and serotype distribution of *Streptococcus agalactiae* in third trimester pregnancy in southern Ghana. BMC Pregnancy Childbirth.

[38] Bobadilla F.J., Novosak M.G., Cortese I.J., Delgado O.D., Laczeski M.E. (2021). Prevalence, serotypes and virulence genes of *Streptococcus agalactiae* isolated from pregnant women with 35–37 weeks of gestation. BMC Infect. Dis..

[39] Furfaro L.L., Nathan E.A., Chang B.J., Payne M.S. (2019). Group B streptococcus prevalence, serotype distribution and colonization dynamics in Western Australian pregnant women. J. Med. Microbiol..

[40] Akpaka P.E., Henry K., Thompson R., Unakal C. (2022). Colonization of *Streptococcus agalactiae* among pregnant patients in Trinidad and Tobago. IJID Reg..

[41] Schindler Y., Rahav G., Nissan I., Madar-Shapiro L., Abtibol J., Ravid M., Maor Y. (2020). Group B Streptococcus serotypes associated with different clinical syndromes: Asymptomatic carriage in pregnant women, intrauterine fetal death, and early onset disease in the newborn. PLoS ONE.

[42] Morozumi M., Wajima T., Takata M., Iwata S., Ubukata K. (2016). Molecular Characteristics of Group B Streptococci Isolated from Adults with Invasive Infections in Japan. J. Clin. Microbiol..

[43] Eskandarian N., Ismail Z., Neela V., van Belkum A., Desa M.N., Amin Nordin S. (2015). Antimicrobial susceptibility profiles, serotype distribution and virulence determinants among invasive, non-invasive and colonizing *Streptococcus agalactiae* (group B streptococcus) from Malaysian patients. Eur. J. Clin. Microbiol. Infect. Dis..

[44] Shabayek S., Vogel V., Jamrozy D., Bentley S.D., Spellerberg B. (2022). Molecular Epidemiology of Group B Streptococcus Colonization in Egyptian Women. Microorganisms.

[45] Imperi M., Gherardi G., Alfarone G., Creti R. (2024). Group B Streptococcus Infections in Non-Pregnant Adults, Italy, 2015–2019. Pathogens.

[46] Teatero S., Ramoutar E., McGeer A., Li A., Melano R.G., Wasserscheid J., Dewar K., Fittipaldi N. (2016). Clonal Complex 17 Group B Streptococcus strains causing invasive disease in neonates and adults originate from the same genetic pool. Sci. Rep..

[47] Plainvert C., Hays C., Touak G., Joubrel-Guyot C., Dmytruk N., Frigo A., Poyart C., Tazi A. (2020). Multidrug-Resistant Hypervirulent Group B Streptococcus in Neonatal Invasive Infections, France, 2007–2019. Emerg. Infect. Dis..

[48] Petca A., Șandru F., Negoiță S., Dumitrașcu M.C., Dimcea D.A., Nedelcu T., Mehedințu C., Filipov M.M., Petca R.C. (2024). Antimicrobial Resistance Profile of Group B *Streptococci* Colonization in a Sample Population of Pregnant Women from Romania. Microorganisms.

[49] Kekic D., Gajic I., Opavski N., Kojic M., Vukotic G., Smitran A., Boskovic L., Stojkovic M., Ranin L. (2021). Trends in molecular characteristics and antimicrobial resistance of group B streptococci: A multicenter study in Serbia, 2015–2020. Sci. Rep..

[50] Shipitsyna E., Shalepo K., Zatsiorskaya S., Krysanova A., Razinkova M., Grigoriev A., Savicheva A. (2020). Significant shifts in the distribution of vaccine capsular polysaccharide types and rates of antimicrobial resistance of perinatal group B streptococci within the last decade in St. Petersburg, Russia. Eur. J. Clin. Microbiol. Infect. Dis..

[51] Quiroga M., Pegels E., Oviedo P., Pereyra E., Vergara M. (2008). Antibiotic susceptibility patterns and prevalence of group B Streptococcus isolated from pregnant women in Misiones, Argentina. Braz. J. Microbiol..

[52] Decoster L., Frans J., Blanckaert H., Lagrou K., Verhaegen J. (2005). Antimicrobial susceptibility of group B streptococci collected in two Belgian hospitals. Acta Clin. Belg..

[53] Da Cunha V., Davies M.R., Douarre P.E., Rosinski-Chupin I., Margarit I., Spinali S., Perkins T., Lechat P., Dmytruk N., Sauvage E. (2014). *Streptococcus agalactiae* clones infecting humans were selected and fixed through the extensive use of tetracycline. Nat. Commun..

[54] Bridley J., Nelson J. (2024). 2024 Nelson’s Pediatric Antimicrobial Therapy.

[55] Flannery D.D., Ramachandran V., Schrag S.J. (2025). Neonatal Early-Onset Sepsis: Epidemiology, Microbiology, and Controversies in Practice. Clin. Perinatol..

[56] WHO Group B Streptococcus (GBS). https://www.who.int/teams/immunization-vaccines-and-biologicals/diseases/group-b-streptococcus-(gbs)#.

[57] Plainvert C., Anselem O., Joubrel C., Marcou V., Falloukh A., Frigo A., Magdoud El Alaoui F., Ancel P.Y., Jarreau P.H., Mandelbrot L. (2021). Persistence of group B Streptococcus vaginal colonization and prevalence of hypervirulent CC-17 clone correlate with the country of birth: A prospective 3-month follow-up cohort study. Eur. J. Clin. Microbiol. Infect. Dis..

[58] Barro C., Salloum M., Lim S., Delputte P., Le Doare K. (2023). Simultaneous carriage of multiple serotypes of Group B Streptococcus: Systematic review and meta-analysis. Vaccine.

[59] Yuan X.Y., Liu H.Z., Liu J.F., Sun Y., Song Y. (2021). Pathogenic mechanism, detection methods and clinical significance of group B *Streptococcus*. Future Microbiol..

